# A novel Arabic tool of cognitive assessment in patients with mild cognitive impairment

**DOI:** 10.1192/j.eurpsy.2022.1670

**Published:** 2022-09-01

**Authors:** S. El Sayed, S. Gomaa, Z. Gomaa

**Affiliations:** Faculty of Medicine-Mansoura University, Department Of Psychiatry,faculty Of Medicine, Mansoura University, Mansoura, Egypt

**Keywords:** THINC-it, COGNITIVE, ARABIC, NOVEL

## Abstract

**Introduction:**

Mild cognitive impairment is one of the commonly reported disorders nowadays in old age individuals,it might represent the prodroma of definite dementia.There is a novel Arabic tool now which could help in the evaluation of cognitive functions in these patients.

**Objectives:**

1-To study the cognitive functions in mild cognitive impairment by a novel Thinc-it tool (Arabic version) 2-To compare between Mini Mental Status Examination ( Standard test) and the novel Thinc-it battery ( Arabic version ) in detection of cognitive dysfunctions in these patients.

**Methods:**

1-The Mini Mental State Examination (MMSE) is a tool that can be used to systematically and thoroughly assess mental status. It is an 11-question measure that tests five areas of cognitive function: orientation, registration, attention and calculation, recall, and language. The maximum score is 30. A score of 23 or lower is indicative of cognitive impairment. The MMSE takes only 5-10 minutes to administer and is therefore practical to use repeatedly and routinely 2-Thinc-it THINC-it® is a screening tool designed to measure cognition and provides important data for an overall evaluation of whether cognitive functioning is impaired,it includes the folloowing tests:

1. PDQ-5D Subjective Questionnaire
2. “Spotter” – CRT game
3. “Symbol Check” – Nback game
4. “CodeBreaker” – DSST game
5. Trails – TMT game

**Results:**

The results of Thinc-it (Arabic version) is statistically correlated to the mean score of Mini Mental state Examination,this means this Arabic version is a valid novel tool for assessment of Cognitive dysfunctions .

**Conclusions:**

Arabic version of Thinc-it can be used in cognitive evaluation

**Disclosure:**

No significant relationships.

